# Late-onset ulnar neuritis following treatment of lepromatous leprosy infection

**DOI:** 10.1371/journal.pntd.0007684

**Published:** 2019-08-19

**Authors:** Trevor Wellington, Christina Schofield

**Affiliations:** Department of Infectious Diseases, Madigan Army Medical Center, Joint Base Lewis-McChord, WA, United States of America; Hospital Infantil de Mexico Federico Gomez, UNITED STATES

## Abstract

Neuritis is a frequent complication of *Myocobacteria leprae* infection and treatment due to the variety of mechanisms through which it can occur. Not only can mycobacterial invasion into peripheral nerves directly cause damage and inflammation, but immune-mediated inflammatory episodes (termed leprosy reactions) can also manifest as neuritis at any point during infection. Treatment of leprosy reactions with thalidomide can also lead to neuritis due to an adverse drug effect. Neuritis can emerge years after initial diagnosis and treatment, although it is most frequently found at time of diagnosis or early into the treatment course. Treatment of neuritis is dependent on high-dose corticosteroid therapy as well as therapy for suspected underlying etiology. Here, we present a case of ulnar neuritis presenting in a patient with lepromatous leprosy four years after treatment of initial infection, with subsequent improvement after corticosteroid burst while maintained on thalidomide therapy.

## Author summary

Leprosy infection is frequently complicated by peripheral neuritis, but is often difficult to distinguish if it is due to persistent infection, an immune-mediated reaction, or adverse drug reaction. In this case, we present a patient with ulnar neuritis occurring four years after multidrug therapy, with improvement with high-dose corticosteroid therapy while remaining on thalidomide treatment.

## Introduction

*Mycobacterium leprae* is a non-motile, acid-fast bacteria that infects its host by invasion of Schwann cells, the principle support cell in the peripheral nervous system. The entry of *M*. *leprae* into peripheral nerves has been thought to cause nerve function impairment independently of the subsequent immune response to infection, although the direct effect of *M*. *leprae* on Schwann cells remains unclear. While *in vitro* studies suggest no loss of Schwann cells by the organism (favoring cell survival), biopsies of human lesions have demonstrated Schwann cell apoptosis [1]. The significance of these findings relative to nerve injury is unclear, as subsequent inflammatory responses, termed leprosy reactions, are thought to be largely responsible for the neurologic manifestations of leprosy infection. The variability of clinical presentation is thought secondary to genetic variability determined by different biological pathways modulated by *M*. *leprae*, as well as the reprogramming of adult Schwann cells and interactions of innate and adaptive immunity [2].

Leprosy reversal reactions are immune-mediated inflammatory complications that occur in treated and untreated *M*. *leprae* infections, affecting 30–50% of all leprosy patients. Two subtypes of reactions are recognized, initially due to their differing clinical presentation and later their suspected immunopathology: type 1 reactions (also referred to as reversal or downgrading) and type 2 (*erythema nodosum leprosum*, ENL) [3]. The neurologic manifestations are secondary to peripheral nerve injury and are characterized by loss of sensation, paralysis, and deformity [4]. These reactions can occur before, during, or after multidrug therapy (MDT) treatment of underlying infection, and can both manifest with neuritis.

Type 1 reactions are cell-mediated reactions characterized by inflammatory changes at sites of mycobacterial infection. Skin changes include the development of tender, erythematous, and swollen lesions at sites of high mycobacterial burden, while affected nerves can have altered sensory and motor function. Type 2 reactions (ENL) are immune-complex mediated reactions with systemic features to include painful, erythematous subcutaneous nodules as well as fever, lymphadenitis, arthritis, iridocyclitis, orchitis, and notably, neuritis. Whereas the recovery rates for type 1 reactions may be as high as 70% if treated within 6 months of onset, ENL may have a chronic or relapsing nature, causes long term neuropathy and subsequent disability. While both subtypes of reactions can cause nerve inflammation and damage, they are thought more common in type 1 reactions but have been found to occur in up to 35% of patients with ENL [4].

The treatment of type 1 reactions relies on corticosteroids (often at high doses for prolonged periods) while continuing to treat underlying *M*. *leprae* infection. Thalidomide is a highly effective treatment for ENL, while corticosteroids remains common primary or additional choice due to restrictions and/or unavailability of thalidomide. In cases of neuritis, corticosteroids are thought to reduce intraneural edema and reduce the inflammatory response to *M*. *leprae* antigens [5]. While MDT provides an effective and standardized regimen for treatment of underlying *M*. *leprae* infection, treatment doses/duration of corticosteroids for leprosy reactions remains dependent on clinical response. Many cases are complicated by reaction relapses in both subtypes, as well as a high degree of a chronic course of ENL. Here, we present a case of steroid-responsive ulnar neuritis occurring in a lepromatous leprosy patient on chronic thalidomide therapy several years after completion of MDT.

## Material and methods

### Ethics statement

Patient study was routine in nature with guideline-directed medical therapy adhered to. No experimental treatment was pursued in treatment of this patient. Consent for publication of case report as well as images was obtained from patient both verbally and in written format, with the patient demonstrating full understanding and agreement with publication and dissemination of clinical course and treatment, with removal of personally identifiable information from case reports as well as images.

Patient information, medical history, and clinical course were obtained via electronic medical records chart review of subspecialty documentation and radiology reports during the period September 2012 through May 2019. All personally identifiable information (PII) was removed to maintain anonymity of patient. Patient images obtained upon informed consent of patient and supplied by authors of this manuscript. The Institutional Review Board was not involved in procedures, given the retrospective and non-experimental nature of this study.

## Results

**Fig 1:**
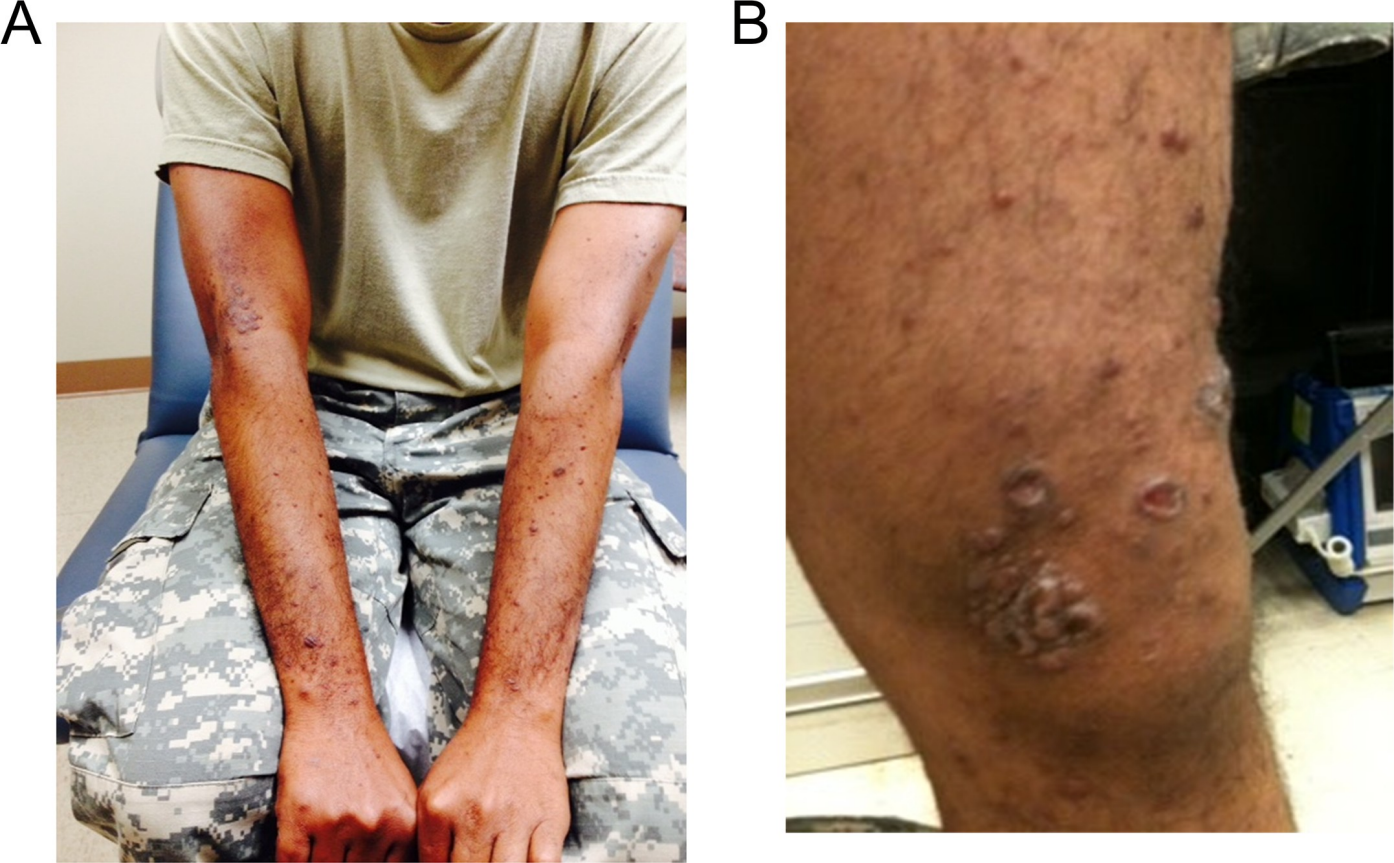
*Erythema nodosum leprosum* (ENL) manifesting as painful, erythematous lesions on patient’s forearms (a) and left knee (b) 24 months into initial treatment course. Lesions resolved with thalidomide treatment but returned upon cessation of therapy.

A 33-year-old Micronesian man with lepromatous leprosy presented with one month of difficulty with writing and right hand/finger numbness in the ulnar distribution, four years after completion of MDT. He had been diagnosed by skin biopsy six years prior and completed two years of MDT with rifampin, dapsone, and clofazimine. His treatment course had been complicated by concomitant development of numerous erythematous and tender lesions on bilateral arms and legs (Fig 1) consistent with ENL beginning one year into MDT. For ENL, he was treated with thalidomide 100mg daily during MDT with several failed attempts to discontinue secondary to recurrence of lesions on his upper and lower extremities (ENL relapse). He had subsequently been restarted on thalidomide 100mg daily after three month thalidomide-free period due to ENL relapse one month prior to presenting with right hand numbness/weakness. Formal nerve conduction studies were obtained of the right arm to elucidate the underlying etiology of his weakness as well as potential burden of nerve impairment, which demonstrated right ulnar neuropathy at the elbow involving focal demyelination and severe (75% - 85%) axonal loss. A right elbow MRI was obtained to assess for presence of nerve compression, which demonstrated focal area of ulnar nerve inflammation within the cubital fossa without mass effect. The patient was diagnosed with ulnar neuritis thought secondary to immune-mediated neuritis related to infiltration of *M*. *leprae* into the nerve and was treated with prednisone taper consisting of 1mg/kg for two weeks followed by 20mg for three months upon demonstration of clinical stability of right hand numbness. Prednisone was then discontinued after completion of a five-week taper. He remained on thalidomide 100mg daily throughout corticosteroid treatment as well as one year after completion of steroid taper, due to his history of frequent ENL relapses. The patient was followed monthly after completion of prednisone taper for monitoring of neuritis, as well as to manage his thalidomide treatment and monitor for ENL relapse. At three months following steroid treatment, he reported no difficulty in gross motor and ADL tasks but continued to have some residual difficulty with fine instrumented tasks with right hand. At one year after completion of the prednisone taper, the patient’s right hand numbness and weakness had resolved, with the patient demonstrating full strength, sensation, and range-of-motion on neurologic examination.

## Discussion

Peripheral neuritis remains a particularly challenging aspect of care for leprosy patients, as it is often difficult to determine whether the etiology of nerve injury is due to infiltration of mycobacteria into peripheral nerves causing damage the subsequent immune response (as seen in both type 1 reactions and ENL), or both [1]. This patient’s neuritis developed substantially late in his clinical course compared to other documented neuritis cases: in cohort study of 78 patients, Raffe et al. report all cases of neuritis occurred either at time of leprosy diagnosis or within the first 12 months of treatment [4]. Given the late onset of this patient’s presentation as well as his frequent ENL recurrences, we feel this patient’s neuritis is most likely consistent as a complication of ENL. The patient had completed MDT approximately four years prior to presenting with new onset right hand weakness, so it is thought less likely due to damage from initial or persistent mycobacteria invasion into the nerve, as this would likely have presented earlier on. While it is unknown the exact trigger of type 1 reactions, they are seen more frequently in the borderline subtype than pure lepromatous subtype. This patient’s leprosy infectious was demonstrated to be lepromatous subtype on skin biopsy at time of diagnosis (to be distinguished histologically from tuberculoid and borderline subtypes). Although nerve function impairment occurring lepromatous patients has been demonstrated to occur independently of leprosy reactions [6], these were cases of early nerve function impairment, occurring around time of diagnosis/treatment of primary infection.

Just as it remains difficult to elucidate the underlying etiology of peripheral neuritis in leprosy patients, management presents its own set of challenges. The treatment for leprosy reactions relies on thalidomide for ENL as well as high-dose steroids for both subtypes of leprosy reactions. ENL remains one of the few indications for thalidomide and requires frequent outpatient monitoring. Corticosteroids present their own set of challenges, as high doses and durations are often requires to achieve clinical stability/improvement. Prednisolone for five months was found superior to three months of treatment, with dosing of 60mg daily being marginally and non-statistically better than 30mg for this duration [7]. ENL cases may require higher doses/durations of steroids as refractory symptoms have been reported in patients on prednisone 60mg for three months [8]. This patient’s stability at three months post-corticosteroid taper suggests that initial treatment with high-dose prednisone at 1mg/kg for two weeks, then three months of 20mg prednisone followed by taper may offer an alternative treatment strategy. Methotrexate also remains an option for patients who cannot tolerate higher doses of corticosteroids or have refractory symptoms despite high doses. Treatment with prednisone 20mg daily with methotrexate 2.5 mg every 12 hours, three times per week demonstrated improvement in neural pain in nine-month period in patients with chronic neuritis secondary to leprosy (regardless of subtype) [5].

Given this patient’s prolonged exposure to thalidomide treatment over several years, it also remains possible that his neuritis is secondary to adverse drug effect of thalidomide, as peripheral neuropathy has been described in patients undergoing thalidomide treatment for leprosy [9] as well as multiple myeloma [10]. These patients demonstrated sensory length-dependent neuropathy presenting as painful paraesthesias or numbness, with greater degeneration correlating to total cumulative dose of thalidomide. The potential etiology for our patient’s neuropathy thus ranges from primary infection effect to leprosy reaction (both type 1 and ENL) to adverse effect from thalidomide. However, the patient’s improvement in right hand function, with full strength, sensation, and range of motion one year after completion of prednisone taper while remaining on daily thalidomide suggest it is less likely to be an adverse effect of thalidomide therapy. While a trial off of thalidomide treatment to observe its effect on his neuritis could be considered, his frequent ENL relapses while off of thalidomide prohibit discontinuation at this time.

This patient’s clinical stability without progression of neuritis after completion of the corticosteroid taper provides an example of late-onset neuritis in leprosy patient, as well evidence for a reasonable treatment approach. Regardless, leprosy reactions, both of which can manifest with neuritis, remain a challenging complication of leprosy treatment that requires diligent monitoring for months to years following initiation of treatment.

## Supporting information

S1 Supporting Information Patient consent form. (PDF)
